# Influence of Limestone Powder as Activator on the Enhancement of Early Mechanical Strength and Durability of High Blending Fly Ash Mortar Cured Under Different Temperatures

**DOI:** 10.3390/ma18225087

**Published:** 2025-11-09

**Authors:** Qingfeng Chen, Weizhun Jin, Jingjing Li, Min Huang, Pengfei Fang

**Affiliations:** 1 College of Civil Engineering, Henan University of Engineering, Zhengzhou 451191, China; chenqingfeng@haue.edu.cn (Q.C.); jianai729505@163.com (J.L.); huangmin_2006@163.com (M.H.); 2 College of Civil and Transportation Engineering, Hohai University, Nanjing 210024, China; 3 College of Civil Engineering, Henan University of Technology, Zhengzhou 450001, China; fangpf_2018@163.com

**Keywords:** limestone powder, fly ash mortar, early mechanical strength, chloride diffusion coefficient, freeze–thaw resistance

## Abstract

The early mechanical strength of high blending fly ash (FA) concrete in the hardening process is extremely low and greatly affected by curing temperatures, which seriously affects the construction speed and durability. To solve this problem, the influence of limestone powder (LS) as an activator on the enhancement of early mechanical strength and durability of high blending FA mortars cured under 5, 20, and 50 °C was investigated. The results reveal that LS can increase the early compressive strength of mortars with 30% FA cured under 5, 20, and 50 °C at 3 d by up to 21.0%, 18.2%, and 21.2%, respectively. Meanwhile, FA mortar with LS cured under 20 °C can achieve a maximum resistance to freeze–thaw cycles and chloride ion erosion, followed by under 50 °C and 5 °C. Under high curing temperatures of 20 °C and 50 °C, the nucleation effect of LS is more conducive to the cement hydration in FA mortar to produce more hydration products and the microchemical activity of LS can facilitate CaCO_3_ from LS to react with calcium aluminate in cement to form calcium carboaluminate (Mc). However, a low curing temperature of 5 °C will significantly reduce the nucleation effect and microchemical activity of LS, causing LS to act more as a filler in FA mortar. LS as an activator can promote more hydration products in FA mortars and make the inner structure of FA mortar denser. LS can greatly reduce the total porosity of FA mortar cured under 20 °C and 50 °C.

## 1. Introduction

Due to the expansion of large-scale construction projects, the usage of cement, as the traditional cementitious material in concrete, is still increasing. However, the production of cement requires high-temperature calcination (around 1450 °C), and for each ton of cement clinker, approximately 0.8–1 ton of CO_2_ is produced, accounting for 7–8% of global anthropogenic carbon emission [1,2]. Meanwhile, the raw materials for cement production mainly consist of limestone and clay [3,4]. The global annual extraction volume exceeds 5 billion tons, resulting in the destruction of vegetation in mines and the imbalance of landforms. Therefore, fly ash (FA), which is environmentally friendly, resource-saving, and low in price, has been widely used in concrete engineering [5,6]. The active silica (SiO_2_) and alumina (Al_2_O_3_) in FA can undergo a secondary hydration reaction with the Ca(OH)_2_ produced during cement hydration, resulting in the formation of AFt and C-S-H [7,8]. Meanwhile, finer FA particles (1–45 μm) can fill pores between cement particles (30–50 μm), thereby reducing the porosity of concrete [9,10]. The FA particles are smooth and spherical in shape, which can act as ball bearings in concrete, thereby reducing frictional resistance between particles and lowering the viscosity of the slurry [11,12]. However, in early hydration stage, the pozzolanic effect of FA cannot be stimulated, and only when hydration has progressed to a certain extent, under the action of sufficient calcium hydroxide, the Ca^2+^, [AlO_4_]^5−^ and [SiO_4_]^4−^ in FA participate in hydration to generate new products of C-S-H and hydrated calcium aluminate. This leads to a lower early mechanical strength of concrete with a large amount of FA. Since the low early strength of high blending FA concrete seriously affects the rapid construction and early safety of concrete structures. Therefore, it is urgent to improve the early strength of high blending FA concrete to meet the demand of concrete construction.

Limestone powder (LS) is a fine powder formed by crushing and grinding natural limestone [13]. Limestone is one of the most widely distributed sedimentary rocks in the Earth’s crust, accounting for approximately 15% of the total mass of the crust [14]. Therefore, the global reserves are abundant, making the acquisition of LS extremely easy and the resources abundant. Compared with industrial by-products such as FA and slag, the production of LS is a physical processing process without high-temperature calcination or chemical reactions. Therefore, it has lower energy consumption. Meanwhile, the energy consumption for producing 1 ton of LS is only 1/5 to 1/10 of that for cement, so the carbon emissions are much lower than those of cement. The addition of LS to concrete offers significant advantages in improving the performance of concrete. LS is usually very fine (mostly at the micrometer level, or even at the nanometer level), which can fill the pores in cement paste and optimize particle distribution of concrete [15,16]. Furthermore, the surface of LS can provide a large number of additional nucleation sites for cement hydration products, reducing activation energy for the crystallization of hydration products and facilitating the faster and more uniform formation and growth of hydration products [17,18]. LS can react with AFm to form monocarboaluminate (Mc) [19,20]. The stability of Mc is higher than that of AFt, and they have a smaller volume expansion. This can reduce the volume deformation caused by the later transformation of AFt, such as in high temperature or dry environments, thereby improving the volume stability of concrete. Based on the advantages of LS in concrete mentioned above, it is expected to be used as an activator to enhance the early strength of FA concrete.

Furthermore, as the scale of construction continues to expand around the world, concrete structures are increasingly being built in harsh climates. Hence, the concrete structures during construction are inevitably subjected to harsh temperatures. However, curing temperature significantly affects cement hydration behavior at the early stage of the hardening process [21,22]. High curing temperature accelerates the cement hydration, but the hydration products tend to be unevenly distributed and easily wrap the unhydrated cement particles, which hinders further hydration of cement clinker [23,24]. However, too low a curing temperature will seriously reduce the hydration rate, resulting in a sharp reduction in hydration products in the early stage, which leads to the reduction in the early performance of cement-based materials [25,26,27]. Moreover, the influence of different curing temperatures on the internal structure of cement-based materials will seriously affect the durability of concrete structures in the later stage, especially the resistance to the freeze–thaw cycles and chloride ion erosion of hydraulic concrete [28,29]. Therefore, the study of early mechanical strength and the later resistance to the freeze–thaw cycles and chloride ion erosion of cement-based materials with high blending FA by LS as an activator under different curing temperatures has an important reference significance for accelerating the early concrete construction and long-term durability of concrete structures.

Due to the lack of research on LS in improving the early mechanical strength and durability of concrete containing large amount of FA under different curing temperatures, the influence of LS as an activator on the early compressive strength and resistance to freeze–thaw cycles and chloride ion erosion of high blending FA mortar cured under 5 °C, 20 °C, and 50 °C was investigated. The compressive strength, freeze–thaw resistance of mortars, and chloride diffusion coefficient were evaluated. The hydration products and microstructure of mortars were characterized by XRD, TG, and SEM. The total porosity of mortars was characterized by vacuum water retention and drying methods. The research on LS as an activator in this work can provide crucial theoretical guidance for enhancing the early mechanical strength and resistance to freeze–thaw cycles and chloride ion erosion of concrete with a high content of FA under different environmental temperatures and has significant practical application value in engineering.

## 2. Experimental Procedure

### 2.1. Materials

P·O 42.5 ordinary Portland cement (OPC) (specific surface area of 327 m^2^/kg and density of 3.06 g/cm^3^), FA of class F (specific surface area of 253 m^2^/kg and density of 2.18 g/cm^3^), and LS (specific surface area of 741 m^2^/kg and density of 2.72 g/cm^3^) were used in this work. Table 1 presents the chemical compositions of OPC, FA, and LS. The aggregate is river sand with a fineness modulus of 1.6~3.7. Superplasticizer (SP) is high performance polycarboxylic acid superplasticizer, which has a solid content of 40% and is purchased from Sobute New Materials Co., Ltd., Nanjing, China.

### 2.2. Specimen Preparation

The mix proportions are listed in Table 2. The ratio of water to binder is set as 0.35. The replacement amount of FA for cement is 30% and 60%. The external additive LS is used as an activator in mortar and the amount is 5% by the weight of total binder. In order to improve the fluidity of mortar, 0.6% SP is added into each proportion.

During the preparation of samples, the mortar was poured in the 40 mm × 40 mm × 40 mm molds to measure compressive strength and conduct freeze–thaw resistance test. Meanwhile, the mortar was poured in the Φ100 mm × 50 mm cylindrical cylinder to test chloride diffusion coefficient. The mortar blocks of OPC, FA60, and FA60LS5 were cured under 20 °C in water bath. Moreover, the mortar blocks of FA30 and FA30LS5 were cured under temperatures of 5 °C, 20 °C, and 50 °C in a water bath.

### 2.3. Test Methods

#### 2.3.1. Compressive Strength

The compressive strength was measured according to Chinese standard GB/T 17671-2021 [30]. The compressive strength was carried out by using mortar blocks and the load was uniformly added at 2400 N/s until broken. The arithmetic mean of three samples was regarded as compressive strength.

#### 2.3.2. Chloride Diffusion Coefficient

After mortar blocks were cured for 180 d, the chloride diffusion coefficient test was conducted by RCM method corresponding to Chinese standard GB/T 50082-2009 [31]. First, the rubber sleeve with mortar blocks were installed in a test tank, and an anode plate was also installed. Then, 300 mL of 0.3 mol/L NaOH solution was injected into a rubber sleeve. Meanwhile, 12 L NaCl solution with a mass concentration of 10% was injected into a cathode test chamber. Next, the anode and cathode of the power supply were, respectively, connected to the anode plate in a rubber sleeve and cathode plate in the test tank. The power supply was turned on, and the voltage was adjusted to 30 V to start the test. During the test, the applied voltage should be adjusted constantly, and the initial current should be recorded. According to the initial current, the duration of the test should be determined. After the test, the mortar block was split into two half cylinders along axial direction by a press, and the split section was sprayed with AgNO_3_ solution with a concentration of 0.1 mol/L. Then, the mortar block is divided into 10 equal parts along diameter section. According to the color change, the distance between color boundary and bottom surface is measured, and the average penetration depth of chloride ions was calculated. Finally, the chloride diffusion coefficient was calculated according to Formula (1), and the average value of three samples was taken as the chloride diffusion coefficient of each group.

(1) $$D_{RCM}=\frac{0.0239\times (273+T)L}{(U-2)t}\left(X_{d}-0.0238\sqrt{\frac{(273+T)LX_{d}}{U-2}}\right)$$

where $D_{RCM}$ is chloride diffusion coefficient; U is absolute value of voltage, V; T is average of initial and ending temperatures of anode solution, °C; L is thickness of test block, mm; $X_{d}$ is average penetration depth of chloride ions, mm; t is duration of test, h.

#### 2.3.3. Freeze–Thaw Resistance

After the mortar blocks were cured for 180 d, the freeze–thaw resistance test was conducted by quick frozen method corresponding to Chinese standard GB/T 50082-2009 [31]. The mass and compressive strength of mortar were measured after every 25 and 50 freeze–thaw cycles, respectively. The mass loss rate was calculated by formula (2). The final compressive strength and mass loss rate of each group were obtained by taking the average value of three samples.

(2) $$W_{n}=\frac{M_{0}-M_{n}}{M_{0}}\times 100\%$$

where $W_{n}$ is mass loss rate, %; $M_{0}$ is mass before freeze–thaw cycles, g; $M_{n}$ is mass after n freeze–thaw cycles, g.

During non-destructive tests of internal damages in cement-based materials, the ultrasonic testing technology can determine whether there are cracks, voids, etc., inside the material by analyzing the changes in propagation time of ultrasonic waves within matrix. In this study, a C60 non-metallic ultrasonic testing instrument was used to measure the propagation time of ultrasonic waves in mortar blocks after freeze–thaw cycles. After measuring ultrasonic propagation time, the relative dynamic elastic modulus (RDM) of mortars was obtained from Formulas (3) and (4) [32]. The final RDM of each group was obtained by taking the average value of three samples.

(3) $$DM=\frac{(1+v)(1-2v)\rho V^{2}}{1-v}=\frac{(1+v)(1-2v)\rho L^{2}}{(1-v)t^{2}}$$

(4) $$RDM=\frac{E_{dn}}{E_{d0}}=\frac{V_{n}^{2}}{V_{0}^{2}}=\frac{t_{0}^{2}}{t_{n}^{2}}$$

where DM is dynamic elastic modulus; V is propagation speed of ultrasonic waves within mortars, m/s; L is length of mortar block, m; ρ is density of mortars, kg/m^3^; v is Poisson’s ratio; $t_{0}$ is ultrasonic wave propagation time before the mortar undergoes freeze–thaw cycles, μs; $t_{n}$ is ultrasonic wave propagation time after mortars undergo n freeze–thaw cycles, μs.

#### 2.3.4. XRD, TG, and SEM

The mortar cured for a certain age was immersed in alcohol to stop the hydration, and then dried in an oven under 50 °C for 24 h and broken up into powders. The hydration products were characterized by XRD and TG tests. The XRD patterns were obtained by Bruker D8 Advance (Bruker AXS, Karlsruhe, Germany) with Kb-filtered Cu Ka radiation under 40 kV and 40 mA. The TG analysis was conducted by STA449F5 Jupiter (NETZSCH Group, Selb, Germany). The powders were heated from 30 °C to 900 °C at 10 °C/min under nitrogen atmosphere. The microstructure of mortars was tested by SEM. The broken mortar blocks were dried under 50 °C for 24 h. Then, the mortar blocks were coated with gold and SEM images were obtained by Zeiss Sigma 300 (CARL ZEISS, Oberkochen, Germany).

#### 2.3.5. Total Porosity

The total porosity of the mortars is obtained by a water absorption test according to ASTM C1585 [33]. First, the mortar with a dimension of 40 mm × 40 mm × 40 mm was subjected to vacuum saturated with water for 24 h and then weighed. Then, the mortar was placed in a 105 °C oven for 24 h and weighed again. The total porosity of the mortars was calculated according to Formula (5). The final total porosity of each group was obtained by taking the average value of three samples.

(5) $$P=\frac{m_{s}-m_{d}}{\rho_{w}V_{m}}\times 100\%$$

where P is total porosity, %; $m_{s}$ is the mortar after being saturated with water, g; $m_{d}$ is the mortar after drying, g; $\rho_{w}$ is density of water, g/cm^3^; $V_{m}$ is volume of the mortar block, cm^3^.

## 3. Results and Discussion

### 3.1. Compressive Strength

**Figure 1:**
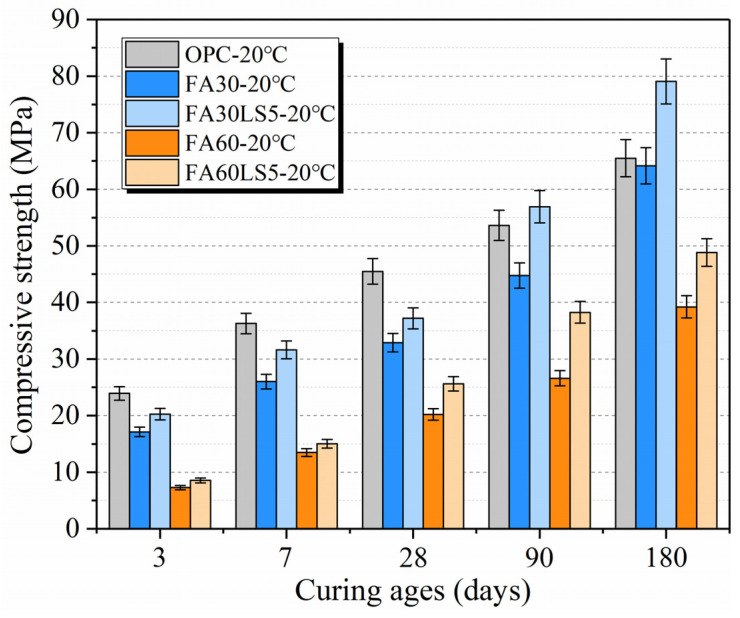
Compressive strength of mortars cured under 20 °C.

Figure 1 shows the effect of LS as an activator on the compressive strength of mortars with large amounts of FA. It can be seen that LS as an activator can effectively improve the compressive strength of mortars with a large amount of FA cured under 20 °C both at early and later ages. LS can enhance the early compressive strength of mortars with 30% and 60% FA by up to 18.2% and 17.7% at 3 d, respectively. Meanwhile, LS can enhance the later compressive strength of mortars with 30% and 60% FA by up to 23.3% and 24.5% at 180 d, respectively. This is mainly attributed to the combined effects of nucleus effect, filling effect, and chemical activity effect of LS on mortar. The smaller LS particles can act as nuclei for the hydration products, providing more surface sites for crystal growth, reducing the activation energy required for crystallization, accelerating the formation of hydration products, and thereby enhancing the early compressive strength of FA mortars [34]. Moreover, compared with cement and FA particles, the LS particles are finer and can be filled into the gaps formed by the accumulation of the cement and FA particles, as well as between the hydration products, thus making the interior of mortars denser and conducive to the improvement of strength [35]. Meanwhile, LS has microchemical activity and can react with Al_2_O_3_ to form monocarboaluminate (Mc), which provides more hydration products with cementitious ability and further improves the strength of mortar [20]. Hence, the filling and chemical activity effects of LS enable LS to continuously enhance the later compressive strength of FA mortar. Furthermore, LS can make the compressive strength of mortar with 30% FA exceed that of ordinary mortar after 90 d but cannot make the compressive strength of mortar with 60% FA exceed that of ordinary mortar. This is mainly because with the increase in age, the pozzolamic effect of FA makes it participate in the secondary hydration of mortar. The active SiO_2_ and Al_2_O_3_ inside the FA react with Ca(OH)_2_ to form more C-S-H, and hydrated calcium aluminate (C_4_AH_13_), and hydrated calcium aluminate (C_2_ASH_8_), which further improves the strength of mortar [7]. This strengthening effect together with the strengthening effect of LS results in a higher strength of mortars than that of ordinary mortar. However, when the amount of LS is too high, the hydration product Ca(OH)_2_ is less, so it cannot provide enough Ca(OH)_2_ to participate in secondary hydration of FA. Therefore, the strength of mortars with 60% FA is less than that of ordinary mortar.

**Figure 2:**
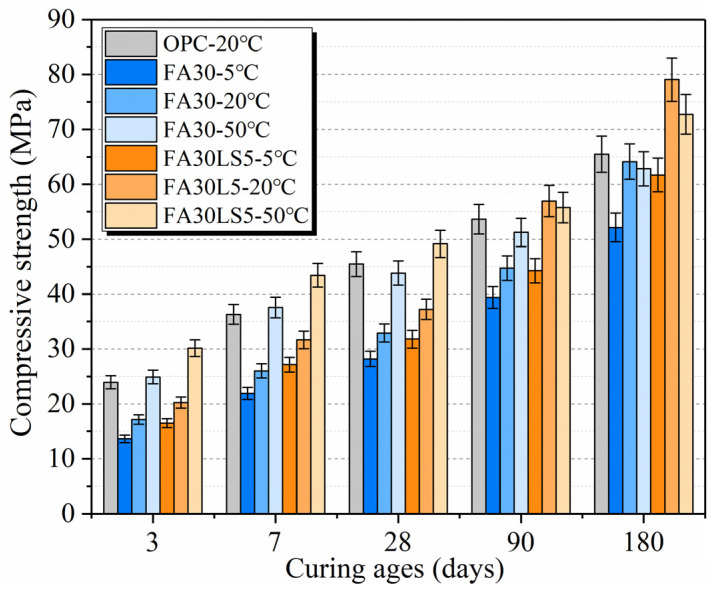
Compressive strength of mortars cured under different temperatures.

Figure 2 presents the compressive strength of mortars cured under different temperatures. Curing temperature has significant effect on FA mortars with and without LS. High temperature curing of 50 °C makes the early compressive strength of mortar much larger than that of 20 °C and 5 °C at the early ages of 3, 7, and 28 d, while 5 °C keeps the lowest compressive strength both at early and later ages. The attributes to the fact that the curing temperature will have an impact on the rate of hydration reaction and the size of hydration products. The high temperature curing of 50 °C accelerates the hydration reaction rate of cement and produces a large amount of hydration products C-S-H and Ca(OH)_2_, which promotes the rapid growth of early strength of mortars [36]. However, too fast hydration reaction is easy to cause uneven hydration products, and the rapidly formed hydration products are easy to be wrapped on the surface of unhydrated cement particles, which leads to the slowdown of cement hydration rate in later stage. Meanwhile, the sharp hydration easily causes the large size growth of hydration products and the accumulation of hydration products is easy to produce large pores [23]. All these reasons make the later strength of mortar under 50 °C less than that under 20 °C. The lower curing temperature of 20 °C makes cement hydration rate slow, which is conducive to full hydration of cement particles and the formation of more C-S-H and Ca(OH)_2_, and the hydration products are evenly distributed. Also, the slow hydration rate makes the particle size of hydration products small, meaning it is easy to form a denser internal structure [29,37]. All these reasons are beneficial to obtaining greater strength of mortar at later ages under 20 °C. However, too low curing temperature of 5 °C makes hydration reaction rate very slow, and produces few C-S-H and Ca(OH)_2_, which makes mortar cementation ability poor, and the internal pores increase, which leads to the mortar curing temperature of 5 °C exhibiting the lowest strength both at early and later stages [36].

Furthermore, LS as an activator can effectively improve the compressive strength of mortars with large amount of FA cured under different temperatures both at early and later ages. LS can increase the early compressive strength of mortars with 30% FA cured under 5 °C, 20 °C, and 50 °C by up to 21.0%, 18.2%, and 21.2% at 3 d, respectively. Meanwhile, LS can increase the later compressive strength of mortars with 30% FA cured under 5 °C, 20 °C, and 50 °C by up to 18.3%, 23.3%, and 15.7% at 180 d, respectively. This is mainly because, regardless of the temperature, the nucleation effect of LS can effectively enhance the early compressive strength of FA mortar, while the filling and chemical activity effects of LS can continuously improve the later compressive strength of FA mortar [38]. Moreover, the compressive strength of FA mortars containing LS under a curing temperature of 20 °C is higher than that under 50 °C from 90 d to 180 d, while the compressive strength of FA mortar without LS under 20 °C does not exceed that under 50 °C until 180 d. This is mainly due to the fact that the fine LS particles can be used as the nucleation site of cement hydration, resulting in the production of more C-S-H and Ca(OH)_2_ at the early age [39]. The increase in Ca(OH)_2_ can promote more FA to undergo secondary hydration, thus producing more hydration products. Therefore, the FA mortar containing LS undergoes almost the same degree of hydration at 90 d under 50 °C and 20 °C. While high curing temperature results in more pores in the inner structure of mortar, which leads to the strength of mortar under 50 °C being lower than that under 20 °C. Furthermore, for the FA mortar without LS, the degree of secondary hydration in FA mortar is low and high curing temperature can accelerate the secondary hydration of FA, which results in the strength of mortars under 50 °C being higher than that under 20 °C. But when the FA mortar undergoes sufficient secondary hydration at age of 180 d, the FA mortar forms more C-S-H and less pores under 20 °C, which results in a higher strength than that under high curing temperature.

### 3.2. Compressive Strength After Freeze–Thaw Cycles

**Figure 3:**
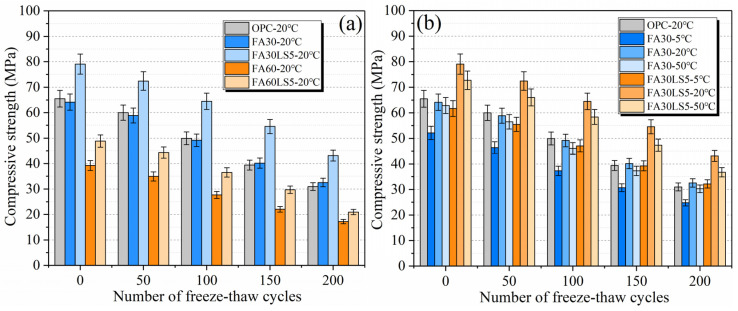
Compressive strength of mortars after freeze–thaw cycles: (**a**) Mortars cured under 20 °C; (**b**) Mortars cured under different temperatures.

Figure 3 displays the compressive strength of mortars after 200 freeze–thaw cycles. From Figure 3a, the compressive strength of FA mortars mixed with LS is still higher than that of FA mortar without LS even after 200 freeze–thaw cycles. After the addition of LS, the compressive strength of mortars with 30% and 60% FA increases by 32.5% and 21.8% compared with that of the mortar containing 30% and 60% FA but without LS after 200 freeze–thaw cycles. This reveals that the addition of LS can greatly improve the freeze–thaw resistance of mortars with large amounts of FA. Furthermore, it can be found that the compressive strength of the mortar with 30% FA is a little higher than that of the mortar without FA after 150 freeze–thaw cycles. This is mainly due to the fact that the secondary hydration of the mortar containing FA continues with time during freeze–thaw cycles and ultimately leads to a gradual increase in compressive strength that eventually exceeds that of the mortar without FA.

From Figure 3b, for FA mortars with or without LS, after 200 freeze–thaw cycles, the compressive strength is still the highest under curing temperature of 20 °C, followed by under 50 °C and 5 °C. This manifests that the freeze–thaw resistance of FA mortars is strongest at 20 °C, followed by 50 °C and 5 °C. Moreover, it can be found that the compressive strength of FA mortar with 5% LS cured under 5 °C is higher than that of FA mortar cured under 50 °C after 100 freeze–thaw cycles and higher than that of the mortar without FA after 200 freeze–thaw cycles. This demonstrates that FA mortar with LS under 5 °C has better freeze–thaw resistance after longer freeze–thaw cycles than the FA mortar under 50 °C and the mortar without FA.

### 3.3. Mass Loss Rate After Freeze–Thaw Cycles

**Figure 4:**
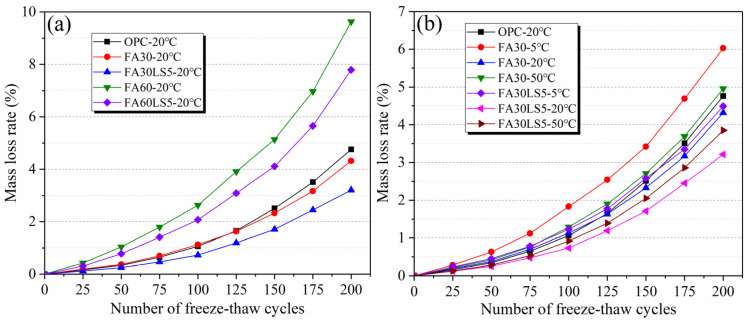
Mass loss rates of mortars after 200 freeze–thaw cycles: (**a**) Mortars cured under 20 °C; (**b**) Mortars cured under different temperatures.

Figure 4 shows mass loss rate of mortars after 200 freeze–thaw cycles. From Figure 4a, the mass loss rate of FA mortar mixed with LS cured under 20 °C is much lower than that of FA mortar without LS after 200 freeze–thaw cycles. After the addition of LS, the mass loss rates of mortars with 30% and 60% FA decrease by 25.7% and 19.1% compared with that of mortars containing 30% and 60% FA but without LS after 200 freeze–thaw cycles. This indicates that the reinforcing effect of LS on FA mortar results in much lower mass loss rate and higher frost resistance of FA mortar. Furthermore, the mass loss rate of the mortar with 30% FA becomes lower than that of the mortar without FA after 125 freeze–thaw cycles. This manifests that the continuous secondary hydration of FA during freeze–thaw cycles enhances the freeze–thaw resistant of mortar matrix.

From Figure 4b, the FA mortars with and without LS can obtain the smallest mass loss rate under curing temperature of 20 °C, followed by 50 °C and 5 °C. This indicates that curing temperature of 20 °C can make the FA mortar obtain the strongest frost resistance, followed by 50 °C and 5 °C. Furthermore, LS can significantly reduce the mass loss rates of FA mortars under different curing temperatures. Even after 175 freeze–thaw cycles, the mass loss rates of FA mortars with LS added under different curing temperatures are lower than that of the mortars without LS and FA. Meanwhile, the mass loss rate of FA mortar mixed with 5% LS cured under 5 °C is less than that of FA mortar cured under 50 °C after 100 freeze–thaw cycles, and it also less than that of the mortar without FA cured under 20 °C after 175 freeze–thaw cycles. This indicates that the promoting effect of LS on the continuous secondary hydration of FA during freeze–thaw cycles greatly enhance the freeze–thaw resistance of FA mortar even under low curing temperature. From the above, it can be seen that at any temperature, LS can enhance the freeze–thaw cycle resistance of FA mortar. This is mainly due to the fact that the tiny LS particles can fill the capillary pores within FA mortar, reducing the porosity and pore size, and minimizing the space where water expands upon freezing. Meanwhile, LS can react with the hydrated product Ca(OH)_2_ to form Mc, thereby filling the voids in the matrix. LS can also stimulate the activity of FA to generate more hydrated products and make the matrix denser.

### 3.4. RDM After Freeze–Thaw Cycles

**Figure 5:**
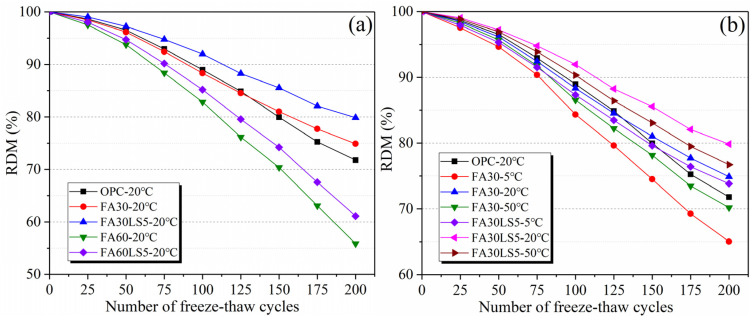
RDM of mortars after freeze–thaw cycles: (**a**) Mortars cured under 20 °C; (**b**) Mortars cured under different temperatures.

Figure 5 displays the RDM of mortars after 200 freeze–thaw cycles. From Figure 5a, the RDM of FA mortar mixed with LS is much higher than that of FA mortar without LS after 200 freeze–thaw cycles. After the addition of LS, the RDM of the mortar with 30% and 60% FA increases by 6.6% and 9.4% compared with that of the mortar containing 30% and 60% FA but without LS after 200 freeze–thaw cycles. This demonstrates that the reinforcing effect of LS on FA mortar results in a much higher RDM and stronger freeze–thaw resistance of FA mortar after freeze–thaw cycles. Furthermore, the mass loss rate of the mortar with 30% FA becomes higher than that of the mortar without FA after 150 freeze–thaw cycles. This manifests in the continuous secondary hydration of FA during freeze–thaw cycles can enhance the matrix and improve the freeze–thaw resistance.

From Figure 5b, FA mortars with and without LS obtain the highest RDM under a curing temperature of 20 °C, followed by 50 °C and 5 °C. This indicates that a curing temperature of 20 °C can make the FA mortar obtain the strongest freeze–thaw cycle resistance, followed by 50 °C and 5 °C. Furthermore, it can be observed that LS can significantly increase the RDM of FA mortars under different curing temperatures and after 175 freeze–thaw cycles, the RDM of FA mortars with LS added under different curing temperatures are higher than that of the mortars without LS and FA. Meanwhile, the RDM of FA mortar mixed with 5% LS cured under 5 °C is higher than that of FA mortar cured under 50 °C after 100 freeze–thaw cycles and also higher than that of the mortar without FA cured under 20 °C after 175 freeze–thaw cycles. This reveals that the promoting effect of LS on the secondary hydration of FA during freeze–thaw cycles significantly enhance the frost resistance of FA mortar even under low curing temperature.

### 3.5. Chloride Diffusion Coefficient

**Figure 6:**
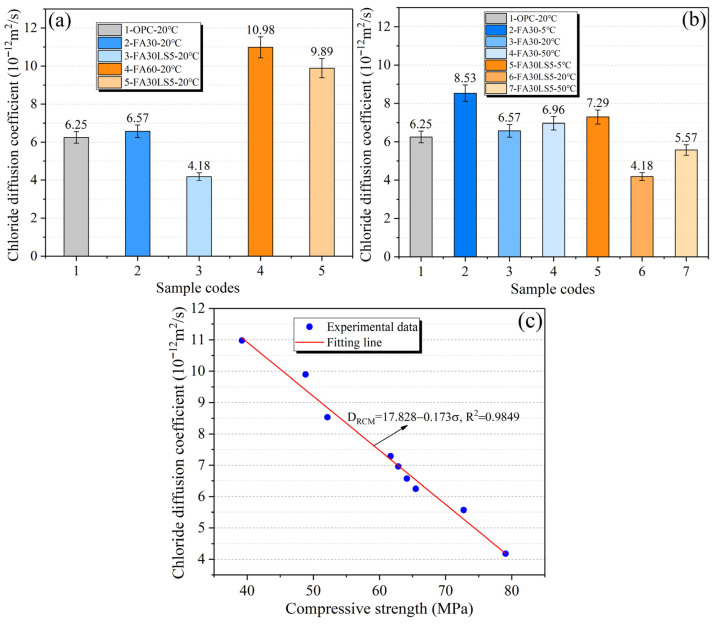
Chloride diffusion coefficient of mortars: (**a**) Mortars cured under 20 °C; (**b**) Mortars cured under different temperatures; (**c**) Relationship between chloride diffusion coefficient and compressive strength.

Figure 6a presents the chloride diffusion coefficient of mortars with large amounts of FA and LS as an activator cured under 20 °C for 180 d. It can be seen that the mortar with 30% FA has just a little larger chloride diffusion coefficient than that of the mortar without FA. It can be seen that the pozzolanic effect of FA makes the chloride ion resistance of FA mortar close to that of ordinary mortar [40]. Furthermore, after the addition of 5% LS into FA mortar, the chloride diffusion coefficient decreases sharply and FA30LS5 obtains a 33.1% reduction in chloride diffusion coefficient compared with OPC. This phenomenon is mainly caused by the filling effect and chemical activity effect of LS can greatly strengthen the mortar matrix. However, the chloride diffusion coefficient of mortar containing 60% FA is much higher than that of the ordinary mortar. The chloride diffusion coefficients of FA60 and FA60LS increased by 75.7% and 58.2% compared with that of OPC. This is mainly due to that when the content of FA is too large, the Ca(OH)_2_ produced by cement hydration will be greatly reduced, so that the pozzolanic activity of FA will be greatly weakened, and the chloride ion erosion resistance of FA mortar will be sharply reduced. Even with the addition of 5% LS, the chloride ion erosion resistance of FA mortar cannot increase too much.

Figure 6b presents the chloride diffusion coefficient of mortars cured under different temperatures for 180 d. For both the FA mortars with or without LS, curing temperature of 20 °C obtains the lowest chloride diffusion coefficient, followed by 50 °C and 5 °C. This is mainly due to the long-term hydration process of mortar. A relatively low curing temperature of 20 °C can promote the cement to produce more C-S-H, and further promote pozzolana reaction of FA, making the mortar have a dense internal structure, resulting in a lower chloride ion diffusion coefficient. However, high temperature curing temperature of 50 °C will lead to uneven distribution of hydration products, resulting in the reduction in hydration products and increase in the internal rough pores of mortar, which leads to the increase in chloride diffusion coefficient of mortar. Furthermore, too low curing temperature of 5 °C will lead to the reduction in hydration products in the mortar and hinder pozzolanic reaction of FA, thus making the internal structure of mortar loose and ultimately leading to the mortar having the maximum chloride diffusion coefficient. In addition, when LS is added, the chloride diffusion coefficient of FA mortar at each curing temperature is much lower than that of FA mortar without LS. This shows that LS plays an excellent role in enhancing the resistance of FA mortar to chloride ion erosion. This is also due to the filling effect of LS and the dense effect of chemical activity on the interior of FA mortar matrix. Meanwhile, curing temperature of 20 °C will be more beneficial to the chemical reaction of LS, which can greatly improve the chloride ion erosion resistance of FA mortar.

Figure 6c displays the relationship between chloride diffusion coefficient and compressive strength. There is a negative linear correlation between chloride diffusion coefficient and compressive strength, which means that the mortar with a higher compressive strength has a lower chloride diffusion coefficient. This is mainly because when the compressive strength is higher, it indicates that there are fewer internal large pores and interconnected pores in the matrix. Chloride ions can only migrate through small and isolated pores, and the diffusion resistance significantly increases. Meanwhile, in the FA mortar with LS added cured under a higher temperature (20 °C and 50 °C), the negative correlation between compressive strength and chloride diffusion coefficient becomes more pronounced. This is mainly due to the fact that at higher temperatures, the nucleation effect of LS and the volcanic ash activity of FA can be fully exerted, which can promote the formation of more C-S-H gels. This helps to increase the compressive strength of the matrix, while also further filling the pores and blocking the intrusion of chloride ions. Furthermore, the high specific surface area of LS can adsorb chloride ions through van der Waals forces, thereby hindering the further diffusion of chloride ions. In addition, the R^2^ is greater than 0.98, which proves the accuracy of this negative correlation. Therefore, if required, the chloride diffusion coefficient of cement-based materials can be estimated from compressive strength.

### 3.6. XRD

**Figure 7:**
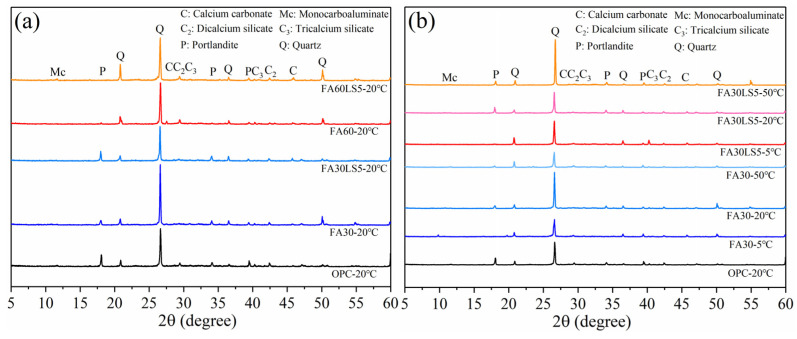
XRD patterns of mortars: (**a**) Mortars cured under 20 °C; (**b**) Mortars cured under different temperatures.

Figure 7a presents XRD patterns of mortars with large dosages of FA and LS as an activator cured under 20 °C for 180 d. It can be seen that compared with the mortar without FA, the Ca(OH)_2_ peaks in FA mortar are significantly weakened. This is due to the depletion of Ca(OH)_2_ by volcanic ash reaction of FA, which weakens Ca(OH)_2_. Furthermore, for the mortar with 30% FA, after the addition of LS, the peak of Ca(OH)_2_ is obviously enhanced, which means that the C-S-H has been improved after the addition of LS. This is mainly due to the nucleation effect of LS can promote cement hydration and produce more Ca(OH)_2_ [41]. In addition, the peak of Mc can be observed in some samples, such as FA60LS5, mainly because the chemical activity effect of LS can react with C_3_A to generate Mc [39]. Due to the small amount of Mc, the peak of Mc cannot be observed in each FA mortar added with LS.

Figure 7b presents XRD patterns of mortars cured under different temperatures for 180 d. It can be seen that curing temperature of 20 °C obtains the strongest Ca(OH)_2_ peaks, followed by 50 °C and 5 °C. This indicates that curing temperature of 20 °C can promote cement hydration to the greatest extent, followed by 50 °C and 5 °C. Furthermore, the peak of Ca(OH)_2_ in FA mortar containing LS cured under 20 °C and 50 °C is higher than that of FA mortar without LS. This is mainly due to the nucleation effect of LS added at higher temperature, which is conducive to the cement hydration in FA mortar to produce more hydration products. In addition, at a low curing temperature of 5 °C, the peak of Ca(OH)_2_ can hardly be seen regardless of whether LS is added or not. This is mainly due to the fact that the too low curing temperature slows down hydration rate, resulting in fewer hydration products. Meanwhile, the volcanic ash effect of FA will consume this small amount of Ca(OH)_2_, resulting in sharp reduction in the amount of Ca(OH)_2_ in FA mortar, and ultimately it will be difficult to observe the peak of Ca(OH)_2_.

### 3.7. SEM

**Figure 8:**
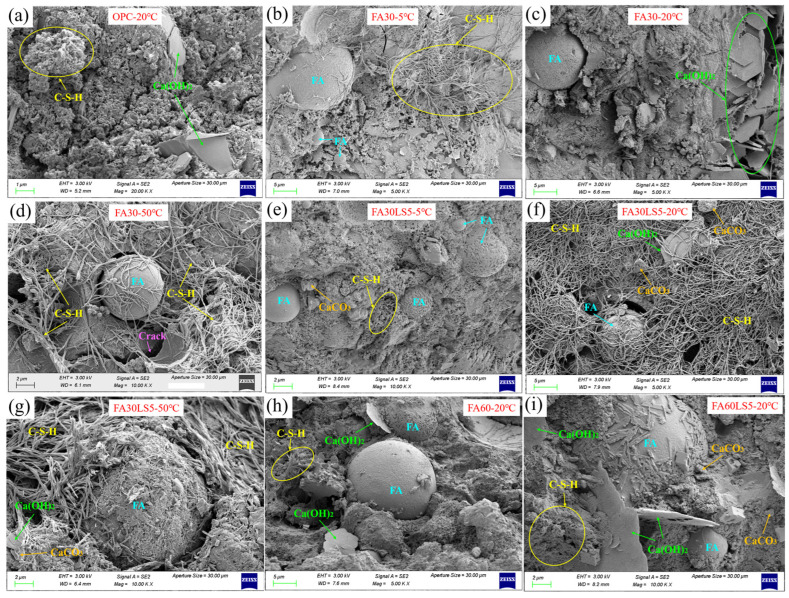
Micromorphology of mortars containing different dosages of FA and LS cured under different temperatures: (**a**) OPC-20 °C; (**b**) FA30-5 °C; (**c**) FA30-20 °C; (**d**) FA30-50 °C; (**e**) FA30LS5-5 °C; (**f**) FA30LS5-20 °C; (**g**) FA30LS5-50 °C; (**h**) FA60-20 °C; (**i**) FA60LS5-20 °C.

Figure 8 presents the micromorphology of mortars with large dosages of FA and LS as an activator cured under different temperatures for 180 d. From Figure 8a, the hydration products of typical flocculent C-S-H and plate Ca(OH)_2_ can be observed in mortars without LS and FA [42,43]. From Figure 8b, FA mortar cured under 5 °C can only form small amount of C-S-H, and the surface of FA is smooth indicating that FA has a low degree of volcanic ash reaction. When FA mortar is cured under 20 °C (Figure 8c), a large number of plate Ca(OH)_2_ is formed, which indicates that hydration reaction of the mortar is sufficient under 20 °C. When FA mortar is cured under 50 °C (Figure 8d), a large number of long fibrous C-S-H can be observed, but at the same time the presence of wide cracks can be observed. This indicates that the hydration degree of mortar under 50 °C is also relatively deep, but because the hydration rate is too fast, cracks are formed inside the mortar, resulting in a loose inner structure. Furthermore, from Figure 8e–g, it can be observed that FA mortars cured under 20 °C and 50 °C can obtain much more hydration products than FA mortar cured under 5 °C. Moreover, after the addition of LS, the nucleation effect of LS can lead to the formation of more hydration products in FA mortar under different curing temperatures compared with FA mortar without LS cured under corresponding temperature. Meanwhile, after the addition of LS, the surface of FA becomes rough, indicating that the addition of LS promotes the pozzolanic reaction of FA, allowing the mortar to produce more hydration products. In addition, smaller particles of LS can be observed in FA mortar, which can be filled into the mortar to make the inner structure denser.

From Figure 8h, it can be observed that the mortar containing 60% FA can form much fewer hydration products than the mortar containing 30% FA. Meanwhile, the smooth and intact FA particles can be clearly observed, mainly because when FA content is high, the dilution effect of FA on cement particles prevents cement hydration and reduces the Ca(OH)_2_, so that a large amount of FA cannot undergo volcanic ash reaction. Furthermore, from Figure 8i, it can be observed that after the addition of finer LS, the hydration products increase due to the nucleation effect of LS. Therefore, FA can react with more Ca(OH)_2_, making the volcanic ash reaction of FA deeper.

### 3.8. TG

**Figure 9:**
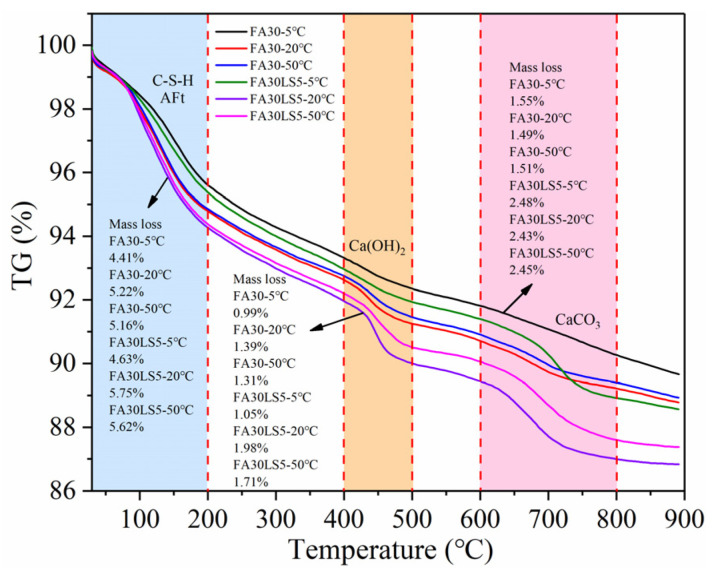
TG curves of mortars cured under different temperatures.

Figure 9 displays the TG curves of mortars cured under different temperatures. The TG curves can be divided into three stages: the first stage ranging from 30 to 200 °C corresponds to the process of C-S-H and AFt losing bound water [42]; the second stage ranging from 400 to 500 °C corresponds to the decomposition of Ca(OH)_2_ and the evaporation of water vapor [36]; the third stage ranging from 600 to 800 °C corresponds to the process of decomposition of CaCO_3_ and the release of CO_2_ [44]. Furthermore, curing temperature can result in the largest mass loss in the first and second stages, followed by 50 °C and 5 °C, which means that curing temperature of 20 °C obtains the most C-S-H, AFt, and Ca(OH)_2_, followed by 50 °C and 5 °C. This is because under room curing temperature, the hydration rate is moderate, which ensures that the ions in the solution have sufficient diffusion time before precipitation, thereby facilitating the continuous cement hydration and formation of more hydration products [23,24]. However, under high curing temperature, due to the rapid rate of hydration reaction, ions do not have enough time to diffuse before the precipitation occurs. This results in the hydrated products already formed encapsulating the unhydrated cement particles, thereby preventing the complete cement hydration and leading to a reduction in the amount of hydrated products [23,45]. Meanwhile, an excessively low curing temperature will result in an abnormally slow hydration rate, thereby causing significant reduction in the amount of hydration products [29]. Moreover, after the addition of LS, the hydration products C-S-H, AFt, and Ca(OH)_2_ all increase. This is mainly due to the nucleation effect of LS, which can lower the nucleation barrier and promote the cement hydration [39]. Also, the incorporation of LS leads to a significant increase in the quality loss in the third stage. This is mainly because most LS cannot participate in hydration reaction and decomposed at high temperatures. Meanwhile, curing temperature of 20 °C can result in the least mass loss in the third stage, followed by 50 °C, and the maximum loss occurs at 5 °C. This is mainly due to the microchemical activity of LS, which can react with the calcium aluminate to form Mc [38]. This reaction is most favorable under room curing temperature, followed by high curing temperature curing. However, at a too low curing temperature, the reaction rate is too slow, resulting in the poorest reaction outcome.

### 3.9. Total Porosity

**Figure 10:**
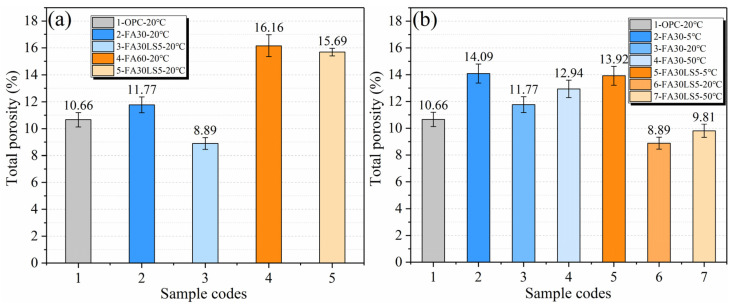
Total porosity of mortars: (**a**) Mortars cured under 20 °C; (**b**) Mortars cured under different temperatures.

Figure 10a presents the total porosity of mortars cured under 20 °C for 180 d. The total porosity of FA mortars after adding LS is significantly reduced, especially when the FA content is 30%, the total porosity of FA mortar after adding 5% LS is 8.89% lower than that without adding LS, which is 11.77%. This is due to the fact that the ultra-fine LS particles can fill the pores inside the mortar, making the mortar obtain a more tightly packed structure, so that the total porosity is greatly reduced [20]. Meanwhile, the cementitious material Mc produced by LS participating in the hydration reaction can further compact the pore structure and contribute to the reduction in the total porosity [46]. Furthermore, when the FA content is 30%, adding 5% LS can make the porosity of FA mortar much smaller than that of the mortar without FA. However, when the FA content is 60%, the effect of LS on the total porosity reduction in FA mortar is limited, and the total porosity of FA mortar cannot be lower than that of the mortar without FA. This is mainly due to the fact that when the replacement amount of FA for cement is too large, the hydration product Ca(OH)_2_ is greatly reduced, and FA cannot carry out sufficient pozzolan reaction, which greatly reduces the hydration product with cementing ability, and ultimately leads to the extremely loose internal structure of FA mortar matrix. Even if LS is added, the improvement effect on pore structure is extremely limited.

Figure 10b presents the total porosity of mortars cured under different temperatures for 180 d. It can be observed that for FA mortar with or without LS, a curing temperature of 20 °C can obtain the minimum total porosity, followed by 50 °C and 5 °C. This is mainly due to the slow hydration reaction at 20 °C and the uniform distribution of hydration products, which results in a relatively denser structure [23,24]. However, under high curing temperature of 50 °C, the hydration products are coarse and unevenly distributed, resulting in large pores [23,45]. Meanwhile, under too low a curing temperature of 5 °C, the hydration rate is extremely slow and too few hydration products can be obtained, leading to a loose internal structure of the mortar, thus having the largest total porosity [29]. Furthermore, the addition of LS greatly reduces the total porosity of FA mortar cured under 20 °C and 50 °C, but has little effect on the total porosity of FA mortar cured under 5 °C. This is mainly because the hydration rate is faster at a higher curing temperature, which can enhance the nucleation effect of LS, increase the hydration products, and make the ash reaction of FA more adequate. At the same time, the chemical activity of LS is higher at higher curing temperature, and more Mc can be produced to fill the internal pores of FA mortar. In addition, the filling effect brought by the finer LS also plays an important role in strengthening the dense structure of FA mortar.

## 4. Conclusions

This work investigates the influence of LS as an activator on the enhancement of early mechanical strength and durability of high blending FA mortar cured under different temperatures. The mortars containing 30% and 60% FA combined with 5% LS were cured under 5 °C, 20 °C, and 50 °C. The compressive strength, freeze–thaw resistance, and chloride ion erosion resistance of mortars were investigated and the microstructure was analyzed. Combined with macroscopic experimental tests and microstructure analysis, the main conclusions are as follows:

(1) LS as an activator can effectively improve the compressive strength of mortars with large amount of FA cured under 20 °C at the early ages. LS can increase the early compressive strength of mortars with 30% and 60% FA by up to 18.2% and 17.7% at 3 d, respectively. Meanwhile, LS as an activator can effectively improve the compressive strength of mortar with a large amount of FA cured under different temperatures at early ages. LS can increase the early compressive strength of mortars with 30% FA cured under 5 °C, 20 °C, and 50 °C by up to 21.0%, 18.2%, and 21.2% at 3 d, respectively.
(2) LS as an activator can significantly enhance the freeze–thaw cycle and chloride ion corrosion resistance of FA mortar. Among them, the FA mortar with LS added under 20 °C curing condition can achieve the maximum resistance to freeze–thaw cycles and chloride ion corrosion, followed by 50 °C curing condition, and the resistance is the smallest at 5 °C curing condition. Meanwhile, the chloride diffusion coefficient of mortars has a negative linear correlation with its compressive strength.
(3) LS as an activator can promote more hydration products of Ca(OH)_2_ and C-S-H in FA mortars. The addition of LS can promote the pozzolanic reaction of FA, allowing the mortar to produce more hydration products. Meanwhile, the nucleation effect and chemical activity effect of LS can be fully exerted under high temperature of 50 °C and 20 °C, thereby generating more hydration products and enhancing the mechanical strength and resistance to freeze–thaw cycles and chloride ion erosion of mortar matrix. In addition, a low temperature of 5 °C will slow down the hydration rate, and the nucleation effect and chemical activity effect of LS cannot be fully exerted.
(4) LS as an activator can be filled into FA mortars, and making the inner structure denser and the Mc produced by the reaction between LS and Ca(OH)_2_ can result in a significant reduction in the total porosity of FA mortars. Meanwhile, the addition of LS can greatly reduce the total porosity of FA mortar cured under 20 °C and 50 °C but has little effect on the total porosity of FA mortar cured under 5 °C.

In this study, through experimental research, it was proved that LS as an activator can significantly enhance the early strength, resistance to freeze–thaw cycles and chloride ion erosion of FA concrete under higher curing temperatures (20 °C and 50 °C), demonstrating significant advantages for engineering application. However, this study still has some obvious deficiencies, especially in the lack of research on the effect of LS as an activator on the resistance of FA concrete to complex environmental erosion. Therefore, subsequent work will conduct studies on the performance of different amounts of LS as an activator against combined chloride-sulfate attack under different curing temperatures to provide more comprehensive theoretical guidance for its engineering application.

## Figures and Tables

**Table 1:** Chemical compositions of OPC, FA, and LS (wt.%).

Materials	CaO	SiO_2_	Al_2_O_3_	Fe_2_O_3_	MgO	SO_3_	K_2_O	LOI
OPC	62.58	20.39	5.43	3.76	1.73	2.28	0.78	3.05
FA	2.47	48.92	36.86	6.79	0.73	0.58	1.07	2.58
LS	56.34	0.37	0.19	0.18	1.69	-	-	41.23

**Table 2:** Mix proportions of mortars (kg/m^3^).

Samples	w/b	Water	Cement	FA	LS	Sand	SP
OPC	0.35	194	554	0	0	1662	3.32
FA30	0.35	194	387.8	166.2	0	1662	3.32
FA30LS5	0.35	194	387.8	166.2	27.7	1662	3.32
FA60	0.35	194	221.6	332.4	0	1662	3.32
FA60LS5	0.35	194	221.6	332.4	27.7	1662	3.32

## Data Availability

The original contributions presented in this study are included in the article. Further inquiries can be directed to the corresponding author.
